# Patterns of physical activity among nursing home residents before and during the Covid 19 pandemic—a systematic observation

**DOI:** 10.1186/s11556-023-00332-5

**Published:** 2023-12-06

**Authors:** Lea-Sofie Hahn, Ansgar Thiel, Dorothée Trüb, Gerhard W. Eschweiler, Andreas M. Nieß, Gorden Sudeck, Annika Frahsa

**Affiliations:** 1 Institute of Sports Science, University of Tübingen, Tübingen, Germany; 2 Interfaculty Research Institute for Sport and Physical Activity, University of Tübingen, Tübingen, Germany; 3 Geriatric Centre, University Hospital of Tübingen, Tübingen, Germany; 4 Department of Sports Medicine, University Hospital of Tübingen, Tübingen, Germany; 5 Institute of Social and Preventive Medicine, University of Bern, Mittelstrasse 43, 3012 Bern, Switzerland

**Keywords:** Covid-19 pandemic, Nursing home, Physical activity patterns, Long-term care facilities, Older adults

## Abstract

**Background:**

The Covid-19 outbreak in spring of 2020 posed an array of challenges for nursing homes, including promoting resident physical activity (PA). Given the diversity of factors affecting resident PA, we explored how activity patterns outside weekly-scheduled structured activities changed during the pandemic and what factors promoted or inhibited PA during the pandemic.

**Methods:**

We conducted systematic direct observations over 823.5 h in eight nursing homes in Southern Germany in 2020 and 2021. *Results:* In 2020, 84.7% of person observation units were classified as sedentary (average activity level: 1.14 MET). In 2021, the percentage increased to 91.6% of observed person units (average activity level: 1.08 MET) (t = 6.947; *p* = .000). According to tree classification, influencing factors of PA included mealtime and daytime in 2020 and 2021, as well as presence of men residents only in 2020 and guided low threshold activities in 2021.

**Conclusions:**

Nursing homes constitute highly sedentary places—an issue exacerbated by access restrictions for external activity experts and significant others as well as behavioural restrictions for residents during the Covid-19 pandemic. Staff could not compensate due to existing time restraints and lack of training in PA promotion. Based on our findings, we recommend future studies to develop feasible and resource-low activities to be integrated into the daily routines of nursing homes.

## Background

During the early part of the Covid-19 pandemic in the spring of 2020, public health authorities in Germany and various other countries [1] published access restrictions and behavioural guidelines for long-term care facilities. Nursing homes were particularly susceptible to high transmission rates from their infrastructural and organisational conditions, like shared bathroom facilities and common living areas. Restrictions aimed to protect older adults in need of care who lived in those settings; however, restrictions also prohibited or limited options for weekly-scheduled structured activities led by external activity experts and guided low threshold activity opportunities, such as strolls with relatives. At the same time, internal staff—who tended to focus on implementing contact restrictions and hygiene measures—did not take over physical activity (PA) promotion [2]. Given the importance of regular PA for the health and physical functioning of older adults, lockdown measures intended to save lives posed life-threatening implications instead [3]. Published annually in Germany, the nursing report determined 70% of nursing home resident relatives described residents as more lonely, depressed, and listless for 2021. Likewise, mental performance and mobility deteriorated [4].

In most cases, PA does not extend beyond everyday activities, such as self-care or moving from one place to another [5–7], yet previous studies identified several factors affecting resident PA levels. *Day of the week* significantly influenced resident PA according to Klenk et al. [8]. For example, on Sundays, residents tend to be less physically active than on other days of the week. Furthermore, PA is often initiated in connection with different *daytimes* (am/pm), especially before and after breakfast, lunch, and dinner (*food intake*). Meals are part of residents monthly fees; therefore, received by all residents [9]. In terms of gender *(men* and *women)*, men seem slightly more active during the day than women residents [6, 10]; more women (66% women) reside in nursing homes than men [11]; and residents spend most of their time among same-gender residents [12]. Since more women reside in nursing homes, more women participate in weekly-scheduled structured activities, which more likely cater to women’s interests [5, 6, 10]. Additionally, *staff or significant others* and their presence influence resident PA. Although residents experience little overall interaction with staff during the course of the day, residents are significantly more likely to be active when staff encourage them [6, 7, 13, 14]. *Activities*—initiated by staff during everyday life—also influence resident PA. Guided low threshold activities include playing with a ball, folding laundry, or setting the table. According to den Ouden et al. [15], they observed residents in those activities in 31% of all observations, with staff often providing support. Sufficient on-site, low threshold activity opportunities are important since many residents cannot independently leave the home to engage in other PA programs in the neighbourhood [13].

Although previous studies identified several factors proven to impact PA among older adults who live in nursing homes, little is known about the impact of the Covid-19 pandemic on PA in nursing home settings. Furthermore, previous studies mostly used accelerometers, interviews, or questionnaires, yet without detailed information about physical and social contexts where PA occurs or monitored group behaviour capabilities [16]. Systematic direct observation constitutes a methodological alternative for studying naturally occurring activity patterns of individuals and groups in specific settings, such as nursing homes, and considering the influence of physical and social environments on PA [17–20] as well as changes in PA behaviour.

In our study, we provide an overview of daily PA—not weekly-scheduled structured activities—of nursing home residents. According to literature, only 54% of women and 34% of men residents participate in weekly-scheduled structured activities [12] that occur on special premises. Previous studies often developed such PA programmes to increase resident PA [5, 21, 22]. However, few residents benefit from weekly programmes offered two to three days a week with low participation or on weekends when no activities take place. To provide a representative overview of resident everyday PA and explore potential changes among their PA from Covid-19 restrictions, we build upon a systematic observation of PA patterns in eight nursing homes in Southwestern Germany between 2020–2021. Our study asked two interrelated research questions:

(1) Outside of weekly-scheduled structured activities, how did PA patterns change among nursing home residents during the Covid-19 pandemic?

(2) What promoting and hindering factors significantly influenced PA patterns among nursing home residents and did factors change from the pandemic?

## Materials and methods

### Study setting

As part of the larger BaSAlt project on PA promotion and counselling in nursing homes *(‘Verhältnisorientierte Bewegungsförderung und individuelle Bewegungsberatung im Setting Altenwohnheim ‘ – ein biopsychosoziales Analyse– und Beratungsprojekt*, funded by the German Federal Ministry of Health 2019–2023, grant no. ZMVI1-2519FSB114), our present study occurred in eight different nursing homes in Southwestern Germany. Institutions differed by environmental context (periphery and urban); management (non-profit institutions), and resident population composition [17]. Living places varied from 33 to 52 and nursing homes contained one (ground level) to three living areas. More women than men lived in all homes; two homes included protected areas for residents with dementia—the reason the number of cognitively impaired residents was higher compared with other homes. Table 1 shows detailed information about nursing home sites.

**Table 1:** Detailed information about nursing home sites

Nursing home	Environmental context	Number of residents	Observed living area
1	Periphery	33	**One living area** dining area (eight tables), TV area (armchairs and sofas), office, kitchen (extra room), floors
2	Periphery	46	**One living area** dining area (four tables and one sofa) with kitchen, floors
3	Periphery	52	**Two living areas** dining areas (three tables each), kitchens (extra rooms), one relaxing area (armchairs and sofas), floors
4	Periphery	48	**Two living areas** dining areas (two to four tables) with kitchen, floors, relaxing area (armchairs)
5	Periphery	40	**Two living areas** dining areas with kitchen (three to five tables), floors
6	Urban	59	**Two living areas** (only observed in 2020) dining areas (five tables), relaxing area (armchairs), floors
7	Urban	46	**Three living areas** dining areas (three tables each) with kitchen (in two areas), floors
8	Urban	39	**Three living areas** dining areas with kitchen (two to four tables), two relaxing areas (armchairs and sofas), floors

### Study design and instrument

We conducted observations guided by the system for observing play and recreation in communities—*SOPARC*—direct observation method [23] to collect data on PA patterns in everyday lives of residents and Thiel et al.'s [19, 20] systematic direct observation tool developed for an observational study on social dynamics of physical (in)activity. Table 2 shows our observation instrument categories.

**Table 2:** Observation instrument categories

**Category**	**Observed Factor**	
**-1-Temporal and spatial-related information**	nursing home date weekday period and area of observation screening time & -ID	Field notes
**-2-Infrastructural-related information**	barriers to PA (+ photo) PA-provoking objects (e.g., balls) activity-enhancing potentials (walking aids) use of activity-enhancing potentials weekly activity plans	
**-3-Person-related information**	gender personal categories (resident, caregiver, significant other) activity categories (passive, sitting, standing, seated rolling, walking +) gait patterns (overlapping, foot to foot, crotch length one foot, crotch length ≥ two feet)	
**-4-Group-related information**	total number of people observed group interactions (verbal, non-verbal) overall activity (%) guided low threshold activities	

We collected *temporal and spatial-related information* (category 1), such as date, weekday, or time, to classify our large amount of data and identify activity hotspots throughout the day or week [24, 25]. To gain knowledge about the indoor *infrastructure* (category 2), we investigated barriers and facilitators for promoting PA (walking aids or PA-provoking objects, such as balls). We developed items concerning *person-related information* (category 3) following McKenzie and colleagues [24, 25]. Observers classified observed persons into personal categories (resident, caregiver, significant other, such as relatives), assigned gender (men, women), and activity categories adapted for nursing home settings (passive, sitting, standing, seated rolling, walking +). We adapted resident gait patterns for the sample [26] into five categories (overlapping, foot to foot, crotch length one foot, crotch length ≥ two feet). To collect *group-related information* (category 4), in line with Thiel et al. [20], we defined all people who entered the observed area as the sample (total number of people observed). To gather further information, we documented verbal and non-verbal (e.g., feeding) interpersonal interactions [24, 25] as well as guided low threshold activities as an important and integral part of daily life in nursing homes. Guided low threshold activities included all PA proposals spontaneously integrated into everyday life by staff. Observers also recorded field notes [25, 27] between screenings and collected weekly activity nursing home schedules to enrich quantitative data with more detailed descriptions of observed PA patterns.

### Data collection

The first observation period ran January to March 2020, the second February to March 2021. We observed various living areas of participating homes to obtain realistic impressions of everyday life. We chose the same observation period both years to avoid seasonal effects. We observed community areas—where meals are served, small activities held, visitors received, or people simply lingered or talked—with a minimum size of 40 m^2^ within nursing homes since a large proportion of residents spent their time there during the day. We did not observe residents in outdoor areas since few residents went outside due to winter season-related cold weather with snowfalls and rain. To capture resident daily PA fluctuations, observation intervals ranged from 10 am to 6 pm (weekdays) and 9 am to 5 pm (weekends). To ensure inter-rater reliability, observers were introduced to all items used in the screening instrument and in a group session, the observers were shown pictures of nursing home residents. Gender, gait patterns and activity categories were discussed together to generate a common understanding. Each observer was accompanied by a developer of the instrument on the first day of data collection and the examples from the group session were available at all times. Nine trained observers collected data with the observation instrument in predefined observation areas in 15 min intervals. In a pilot phase, developers tested the instrument to identify and address problem areas as well as set observation intervals. Intervals were defined by 15 min since nursing homes tend to be low-activity settings [5–7]. Every 15 min, observers overlooked areas and documented all PA-related information with the screening instrument (Table 2). Between screenings, observers wrote fieldnotes about special incidents.

Table 3 provides information about screening days, number of screenings, observation hours, and person observation units in 2020 and 2021. Since the same persons were monitored several times a day, an enormous number of person observation units resulted. In 2020, on-site observations stopped after 34 days from the Covid-19 outbreak and regulations in Germany limiting access to nursing homes for external people.

**Table 3:** Overview of observations in 2020 and 2021

Year	2020	2021	Total
**Observation days**	34	77	111
**Screenings**	800	2494	3294
**Observation hours**	200	623.5	823.5
**Person observation units**	8.454	22.598	31.052
**Resident observation units**	6.153	18.697	24.850

For statistical analyses, we considered the following factors, derived from the existing literature [5–10, 12–14]:

**Table:** 

(1) Day of the week (weekday/weekend) (2) Food intake (mealtime/no mealtime) (3) Men residents (present/not present) (4) Women residents (present/not present)	(5) Staff or significant others (present/not present) (6) Daytime (morning [am]/afternoon [pm]) (7) Activities (guided low threshold activity/unstructured being)

To compare PA, regardless of the total number of people in the observed area, residents were classified into five activity categories. For data evaluation, each activity category was assigned a MET value (metabolic equivalent unit) according to existing literature (Table 4) [28–32]. Overall, we selected rather low MET values (0.95–2.6) for data analysis since residents tended to perform all activities very slowly and with low energy consumption [29].

**Table 4:** MET values of the activity categories

Activity category	MET value
Passive	0.95
Sitting	1.0
Sitting rolling	1.5
Standing	2.0
Walking +	2.6

We defined *passive* (0.95 MET) as lying down or sleeping [28]. We rated *sitting* (1.0 MET) as resting energy expenditure during quiet sitting [29]. *Sitting rolling* (1.5 MET) described moving around independently in a wheelchair using legs but not arms. Elsewhere, the activity was rated as predominantly sedentary, rarely physically active and thus the limit for sedentary behaviour [30, 32]. We defined *standing* (2.0 MET) as standing independently with (e.g., staff, cane) or without help [30], and we rated *walking* + (2.6 MET) as normal walking (on level surface) [31].

### Data analysis

We performed statistical analyses supported by IBM SPSS Statistics 25. For data analysis, due to the high fluctuation of residents, we first calculated t-tests for independent groups to investigate the impact of possible influencing factors on resident PA before and during the Covid-19 pandemic. Additionally, we applied a Bonferroni correction to counteract the problem of erroneously rejecting a null hypothesis from calculating multiple comparisons. To check the practical relevance of differences, we calculated effect sizes of differences using Cohen’s d.

Second, to analyse interaction effects between predictors and differentiate the most pronounced contrast groups concerning PA, we carried out a classification tree analysis (CTA) to identify contrasting groups of PA and test influencing factors for possible interaction effects [33–35]. We used the Exhaustive CHAID algorithm ('Exhaustive Chi-squared Automatic Interaction Detector') for its possibility of a categorial merging for each predictor variable until only two categories remain for each predictor [35]. As a specific ‘stopping rule’ for the analysis, the significance level for splitting nodes and merging categories was set at *p* = 0.05. The depth was set at three and the minimum number of cases in parent nodes was set at 100 and 50 for child nodes. We calculated reliability measures using the risk estimate of misclassification (variance within the nodes). The quality of a tree model was calculated via the explained variance of the tree (variance between the nodes).

Third, we transcribed handwritten qualitative field notes and systematically scanned them for relevant aspects (MAXQDA, 2018) to interpret tree analysis results. We used qualitative data to contextualise and enrich quantitative data.

## Results

We next present results regarding (1) the development of resident PA during the Covid-19 pandemic; (2) different factors influencing PA before and during the Covid-19 pandemic; and (3) field notes to complement results with examples from resident everyday life.

### Development of residents’ PA from 2020–2021

Table 5 shows daily activity differences depending on several influencing factors. In 2020, 84.7% of residents spent most of the day sedentary (average activity level: 1.14 MET). In 2021, the percentage of sedentary residents increased further to 91.6% (average activity level: 1.08 MET) (t = 6.947; *p* = 0.000). Our more detailed analysis shows a significant decrease for most categories—with small effect sizes— on resident PA in 2021 compared with 2020.

**Table 5:** Differences in daily activity depending on several influencing factors in 2020 and 2021

Independent variables	Activity 2020 [MET]	Activity 2021 [MET]	Comparison 2020/2021	Cohen´s d
*Day of the week*				
Weekday	1.12	1.08	t = 4.586 *p* = .000*	0.225
Weekend	1.11	1.10	n.s	
*Food intake*				
Mealtime	1.09	1.06	t = 3.070 *p* = .002*	0.206
No Mealtime	1.13	1.09	t = 3.203 *p* = .002*	0.169
*Men residents*				
Present	1.11	1.08	t = 3.428 *p* = .001*	0.158
Not present	1.14	1.07	n.s	
*Women residents*				
Present	1.12	1.08	t = 4.430 *p* = .000*	0.184
Not present	1.00	1.23	n.s	
*Staff or significant other*				
Present	1.12	1.08	t = 4.671 *p* = .000*	0.210
Not present	1.10	1.09	n.s	
*Daytime*				
Morning (am)	1.09	1.06	n.s	
Afternoon (pm)	1.14	1.09	t = 3.531 *p* = .000*	0.188
*Activities*				
Guided low threshold activity	1.12	1.06	n.s	
Unstructured being	1.12	1.08	t = 3.713 *p* = .000*	0.159

n.s. = not significant; *Significance level after Bonferroni correction: *p* = 0.05/7 = 0.007

### Sedentariness and PA-related contrast groups

**Fig. 1:**
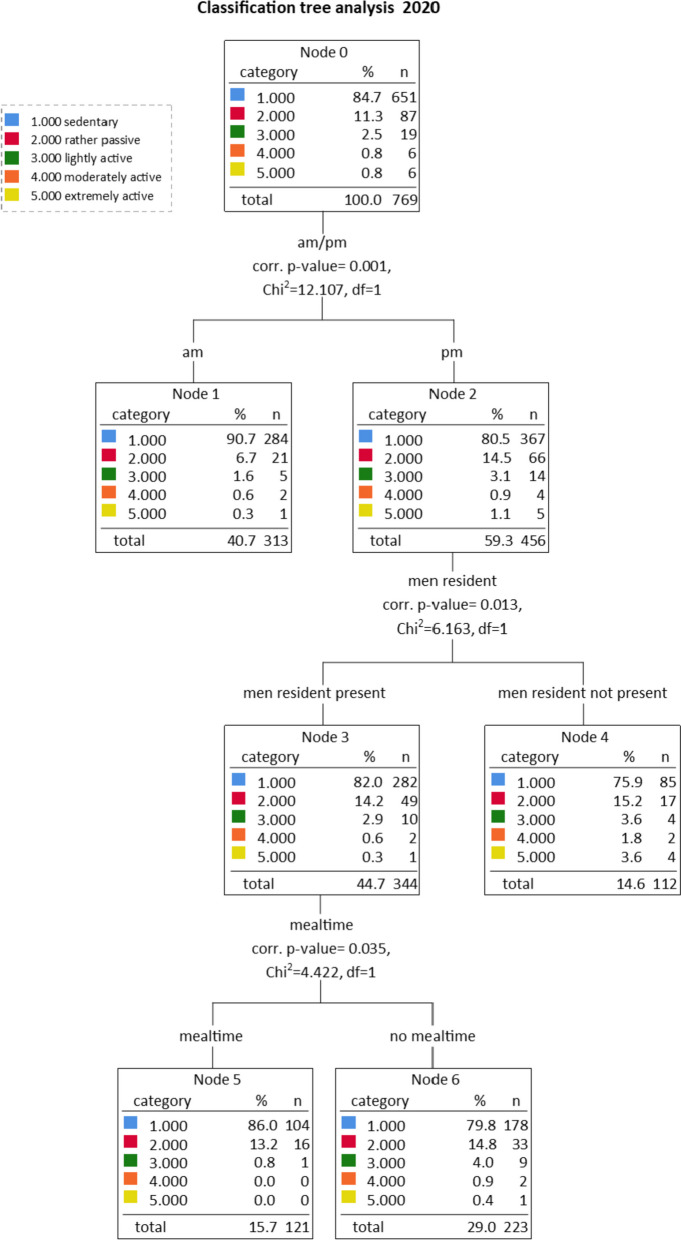
Classification tree of predictors associated with physical activity (PA; 2020). PA levels of groups 1 = sedentary; 2 = rather passive; 3 = lightly active; 4 = moderately active; and 5 = extremely active

**Fig. 2:**
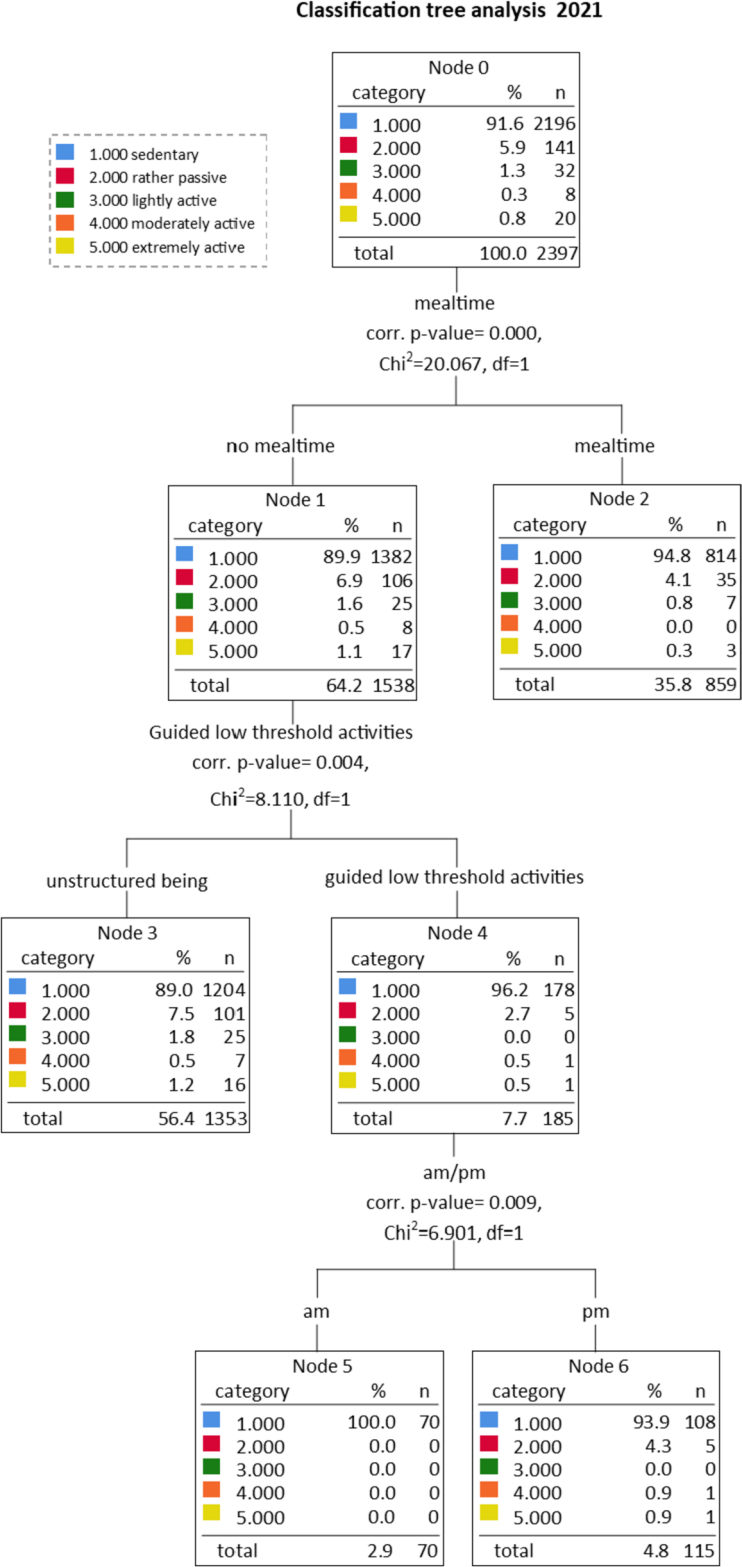
Classification tree of predictors associated with physical activity (PA; 2021). PA levels of groups: 1 = sedentary; 2 = rather passive; 3 = lightly active; 4 = moderately active; and 5 = extremely active

We depict tree models for 2020 and 2021 in Figs. 1 and 2. The activity levels of the classification tree analysis (*sedentary, rather passive, lightly active, moderately active, extremely active*) range from 0.95 to 2.6 MET. For a representative calculation and presentation of the influencing factors, all activity levels cover a range of 0.33 MET to be identical. Residents were significantly more inactive in 2021 (91.6% of residents exhibited predominantly sedentary behaviour) than in 2020 (84.7%). In 2020, 90.7% of residents in the most inactive cluster (node 1, 2020) were sedentary, while in 2021 all residents (100%) were inactive (node 5, 2021). For clusters representing the highest activity, the picture is similar. In 2020, 24.1% of residents in the most active cluster were physically active (node 4, 2020); in 2021, only 11.0% (node 3, 2021).

In detail, the classification tree shows interaction effects between predictors. The first level of the classification tree depicts the factor that explains the largest amount of variance among resident PA— *daytime* (am/pm) in 2020 and *food intake* (mealtime/no mealtime) in 2021 (temporal-related information). At the second level, data shows that in 2020, PA during the afternoon was significantly lower if men residents were present, while in 2021, residents were more active outside mealtime when they could use their time freely (temporal-, personal- and group-related information). At a third level, in 2020, PA in the afternoon was particularly low during mealtimes if men residents were present. In 2021, there was no observable PA at all in the morning outside of mealtime when guided low threshold activities took place (temporal-, personal- and group-related information).

**Fig. 3:**
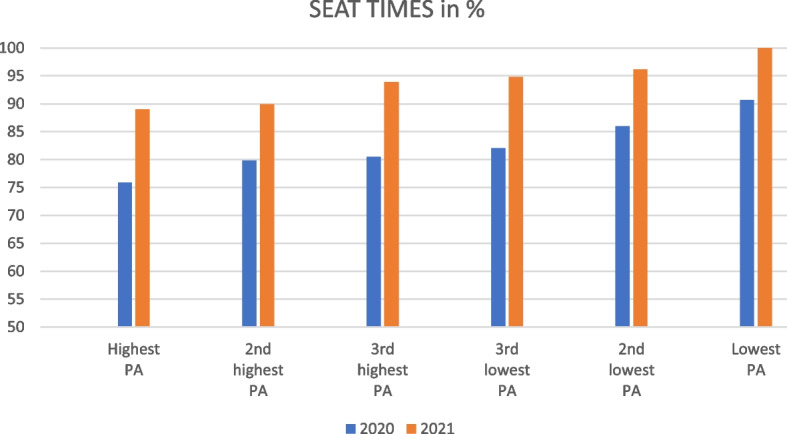
Physical activity clusters directly comparing 2020 and 2021

Figure 3 depicts PA clusters in a direct comparison between 2020 and 2021. In 2021, sitting times clearly increased in all clusters; thus sedentary behaviour appears much more pronounced during the pandemic than before.

### Contextualisation with qualitative data from field notes

Although weekly-scheduled structured activities took place in unobserved areas, on average only eight residents participated. In contrast, low threshold activities in observed areas were initiated and partly guided by staff, which potentially covered all residents in the common areas. These activities happened spontaneously and only when time was available. For instance, “According to the weekly schedule, only few weekly-scheduled structured activities take place (e.g., bowling), but staff often initiates guided low threshold activities in the observation area, such as haptic games, singing, or quizzes” (Field notes, 2021).

As expected, mealtime was associated with physical inactivity—in 2021 even more than in 2020. Residents were hardly involved in any meal-related activities, usually not setting or clearing the table. For example, an observer recorded in field notes, “Staff hands out the food while residents sit at the tables and wait” (Field notes, 2021). When relatives were allowed to spend time in shared areas, they also contributed to resident inactivity by “often visit[ing] at mealtime and sit[ting] at the table to assist residents with eating” (Field notes, 2020). However, it was only observed in 2020, when relatives were allowed to spend time in shared areas. Since “Many relatives come to visit and go directly to the resident's room, as they are not allowed to stay in the shared areas” (Field notes, 2021), PA during mealtimes happened only exceptionally with rather mobile residents. For example, “Normally, staff members start setting the table for lunch, sometimes a resident is helping. After the meal, the staff collects the dirty dishes. Sometimes, a staff member cleans up with residents and has them wipe down the tables” (Field notes, 2021).

Field notes also indicated the relevance of meals as orientation points for PA as many residents come to the dining area long before meals are served: “The observed area fills up about 30 min before lunch and many residents come to the dining area independently (with and without walking aids). Outside of mealtime, only a few residents walk through the area” (Field notes, 2021).

Social interactions played a relevant role in these physically active situations, both in 2020 and 2021. For example, “Two women residents chatted after they walked to each other. […] Some residents go outside together for a walk” (Field notes, 2020); or “* …*residents are often pushed into the observed area long before meals. Even those who can walk independently often arrive early. […] Especially during the pandemic, residents seem to crave social contact and therefore come out of their rooms more often to go to the shared areas” (Field notes, 2021). However, social interactions ‘in motion’ happened more often when Covid-19-related restriction rules were softened and mostly observable when only women were present. Not least, these interactions often occurred spontaneously, for example when two residents met each other and started to talk while walking through the corridors together.

The women residents walk to the women’s club meeting on the first floor. […] A resident is picked up by an external person, and they walk through the corridors […] Two women residents talk about their strolling plans after coffee. (Field notes, 2020)

During lockdown periods, visits—if permitted at all—only took place in resident rooms or outside the nursing home. However, such activities happened much less frequently in 2021 than in 2020. Activities with visitors in community areas came to an almost complete halt during lockdowns. For instance,

A resident is picked up by a visitor for a walk. […] A relative goes to his mother's room and tells the observers that he is unfortunately only allowed to see her in her room and not in the shared areas anymore. (Field notes, 2021)

## Discussion/conclusion

To represent activity in everyday life, we analysed PA patterns of nursing home residents outside weekly-scheduled structured activities just before and during the Covid-19 pandemic. We found a decrease among residents with already low active time from 2020 to 2021; we primarily linked the decrease to behavioural and access restrictions due to the Covid-19 pandemic. We confirmed previous studies that already described a general trend towards sedentariness in nursing homes [5–7]. We also identified factors promoting and hindering PA in this setting [5–13] and showed resident PA is influenced by temporal-related factors, yet also personal- and group-related factors.

### The influence of daytime and guided low threshold activities on PA

According to our classification tree, daytime significantly influenced resident PA with higher inactivity in the morning than in the afternoon. However, daytime is not the decisive factor when looked at in more detail. Before the pandemic (2020), inactivity in observed areas resulted from some residents attending weekly-scheduled structured activities two to three days a week. Here, we even observed a lower participation rate than in other studies, as on average only eight residents per session took part in the weekly-scheduled structured activities such as gymnastics, bowling, or painting [12]. During the pandemic (2021)—with hardly any weekly-scheduled structured activities—low threshold activity opportunities in everyday life became all the more important. According to our classification tree, since external activity experts were not allowed in homes, an enormous amount of inactivity occurred, although staff offered low threshold PA. For staff with limited expertise in PA promotion, complex activations were not possible and mostly simple activities—partly unrelated to PA, such as working on crossword puzzles—in sitting positions took place.

### The influence of mealtime on PA

We found the most dynamic times of day to be directly before and after meals. Meals prove to represent fixed points in resident daily routines, which aligns with other studies [2, 7, 9]. Studies considered mealtime as an activity-provoking highlight of the day, not least because meals are usually served outside private rooms, hence, mealtimes force residents to leave their private rooms and make their ways to dining areas [2, 7, 9].

Low threshold activities related to meals provide new opportunities to encourage residents to be physically active outside weekly-scheduled structured activities. In our study, we observed residents in some participating homes taking over tasks, such as handing out food or setting tables. Even more effective when supported by staff, spontaneous activities provide possible positive impact on resident PA [6, 13, 15]. Spontaneous activity opportunities are possible without investing a large amount of time and personnel, not least of all because residents—as Hoppe [14] also found—show willingness to take over household activities if asked by staff.

### The influence of nursing staff and other social contacts on PA

All homes in our study offered various weekly-scheduled structured or guided low threshold activities to slow down the physical and mental decline of residents prior to the pandemic. During the lockdown, weekly-scheduled structured activities were reduced or even completely suspended, not least from access restrictions for external activity experts. Even if weekly-scheduled structured activities could not be compensated by nursing staff [2], they still made an effort to offer guided low threshold activities during the day. Here, we cannot confirm Hoppe’s [14] findings that nursing homes neglect activities, yet we support not solely relying on external activity experts for PA programmes, since it possibly leads to limitations during pandemics.

Generally, although staff members can positively influence resident PA, lack of time often hinders them [14], as well as lack of competencies for offering PA promotion [2]. Employing PA professionals in-house—which is usually not done in nursing homes—addresses such deficiencies [13, 14]. Furthermore, staff should focus even more on motivating inactive residents, and not only focus on those who are already very active [13]. Since PA in observed areas mostly occurs ‘in motion’ between residents and from time to time with visitors as well, another option would be including significant others to promote PA, while socially interacting with residents since it can stimulate socialisation and improve quality of life [6].

### Strengths and limitations

To our knowledge, our study is the first to systematically observe nursing home resident PA patterns before and during Covid-19. Our combined quantitative and qualitative observations allowed for estimating PA in different everyday life situations and contextualising (in)activity by capturing social, temporal, and personal characteristic activity patterns. Yet, our study also has limitations. Even if gender seems a significant influencing factor on resident PA, the result requires critical reflection. The gender distribution in our study is comparable to the distribution reported in previous studies [11]. Men were observed as less likely to participate in spontaneously-initiated low threshold activities, such as setting the table or folding laundry. Some gender-related findings possibly stem from the presence of almost no men in participating nursing homes, not from a possible activity hindering influence of men. Considering the intensity of PA, a MET value of 1.0 was generally assigned to the category *sitting* without distinguishing between active and passive sitting. Thus, based on our data analysis, categorising sitting behaviour generally as inactive behaviour could lead to an overestimated sedentariness because sitting does not necessarily mean doing nothing physical. In future research, we recommend *sitting* further differentiated into *active* and *passive*. Another limitation involves the difference in observation days between 2020 and 2021 possibly affecting our results. In 2020, observations stopped early from Covid-19 pandemic restrictions. Nevertheless, we observed the same residential areas in both years, only for fewer days in 2020. A final limitation involves direct observation not covering all areas of participating homes. We did not observe rooms where weekly-scheduled structured activities occurred with the screening instrument, yet we recorded activities in field notes. However, only a few—not most—residents participated in activities two to three days a week.

## Conclusions

By identifying promoting and hindering factors for resident PA and PA pattern effects from the pandemic, we provide insights into everyday contexts of PA in nursing homes. Even before the Covid-19 outbreak, sedentary behaviour dominated during observations. Cancelled weekly-scheduled structured activities influenced PA behaviour to some extent, even though on average only eight residents participated. It once again underlines the importance of guided low threshold activities since they can be integrated into everyday life without much effort and performed ‘in motion.’ Nevertheless, even these activities could partially not be performed during the pandemic due to hygiene measures and led to an increase of sedentariness in everyday life.

We suggest findings from our study could be used to integrate feasible and resource-low activities into nursing home daily routines. In the long-term, more active everyday life can slow down the physical and mental decline of residents; thus improving quality of life and autonomy with lower needs for care.

## Data Availability

The datasets generated for this study can be requested via email to the corresponding author.

## Abbreviations

PA: Physical Activity
MET: Metabolic Equivalent Unit
CTA: Classification Tree Analysis
CHAID: Exhaustive Chi-squared Automatic Interaction Detector
